# Vibrio alginolyticus: A Rare Cause of Otitis Externa off the Coast of Florida

**DOI:** 10.7759/cureus.61524

**Published:** 2024-06-02

**Authors:** Anwar A Khan, Benjamin K Linkous, John T Lanza

**Affiliations:** 1 Medical School, Florida State University College of Medicine, Tallahassee, USA; 2 Otolaryngology, ENT Allergy and Associates, Fort Pierce, USA

**Keywords:** climate changes, halophilic bacteria, acute otitis externa, antibiotic susceptibility testing, chronic otitis externa, vibrio alginolyticus

## Abstract

*Vibrio alginolyticus*, a gram-negative marine bacterium, poses significant health risks through various infections transmitted via contaminated seawater or seafood consumption. This case report details a 42-year-old male presenting with chronic seropurulent discharge from his ear, ultimately diagnosed with otitis externa caused by *V. alginolyticus*. Examination findings and antibiotic sensitivity testing informed the treatment strategy, leading to a successful resolution. The increasing incidence of *V. alginolyticus *infections, particularly in warm coastal water, necessitated heightened clinical awareness and appropriate management. As global temperatures rise, proactive measures including patient education and accurate diagnosis become crucial in preventing disease progression and complications associated with *V. alginolyticus *infections.

## Introduction

*Vibrio alginolyticus* is a gram-negative marine bacterium that can cause eye, ear, and wound infections [1]. Infection in humans typically occurs through contact with contaminated seawater or consumption of raw or undercooked seafood [2]. Symptoms include diarrhea, abdominal cramps, vomiting, fever, and skin lesions if infection enters through a wound [2,3]. There have been a few reports of *V. alginolyticus* causing otitis externa in patients with recent exposure to infested seawater, primarily limited to waters around Europe or the Mediterranean in the warmer times of the year [4,5]. The suspected pathogenesis is that the strain exerts cytotoxic effects on keratinocytes and HEI-OC1 cells by inhibiting cell proliferation and migration and inducing apoptosis and cell death [6]. The incidence of *V. alginolyticus* in all disease states is increasing with 1,331 infections reported from 1988 to 2012 in the United States [2,7]. Approximately 17 cases of *V. alginolyticus-*induced otitis externa are reported annually in the United States, with *Pseudomonas *species contributing to 480,000-1,711,200 cases (20%-71.3%) annually and *S. aureus *estimated to be responsible for up to 960,000 cases (40%) annually [2,8,9]. Given the increasing temperature of coastal seawater, the epidemiological tendencies of *Vibrio alginolyticus *should be further investigated.

## Case presentation

A 42-year-old male presents to the clinic with a one-year history of seropurulent discharge from his left ear. He does not recall an inciting event but states that he swims in the warm coastal waters of southeast Florida often. He states that the drainage has been constant for the last year with moderate intensity. The patient denies any hearing loss, otalgia, tinnitus, vertigo, surgery, or trauma. He has not previously sought or received medical care, taken any medications, or had any additional modifying factors.

Examination of the external ear canals and tympanic membrane with a microscope reveals bloody and purulent drainage traversing across the canal and dried blood covering the tympanic membrane. Examination of the nasal cavity showed mucosal inflammation with no other abnormalities noted on physical exam. A culture was taken of the drainage. The patient was prescribed oral amoxicillin 875 mg-potassium clavulanate 125 mg tablet twice a day for 14 days (patient instructed to take prescription with food and probiotics). His culture grew *Vibrio alginolyticus*, and his otitis externa had resolved by his three-week follow-up. Antibiotic sensitivity testing was conducted, revealing that this strain exhibited sensitivity to amoxicillin/clavulanate (with a minimum inhibitory concentration (MIC) ≤ 2.0 ug/mL), ceftazidime (MIC ≤ 1.0 ug/mL), levofloxacin (MIC ≤ 0.12 ug/mL), and trimethoprim/sulfamethoxazole (MIC ≤ 20.0 ug/mL) (Table 1).

**Table 1:** Antibiotic sensitivity profile of Vibrio alginolyticus MIC: Minimum inhibitory concentration

*Vibrio alginolyticus* sensitivity report		
Medication	Interpretation	MIC (ug/mL)
Amoxicillin/clavulanate	Susceptible	≤2.0
Ceftazidime	Susceptible	≤1.0
Levofloxacin	Susceptible	≤0.12
Trimethoprim/sulfamethoxazole	Susceptible	≤20.0

## Discussion

Similar to other halophilic marine *Vibrio* species, *V. alginolyticus* has been identified as pathogenic to humans, causing serious seafood poisoning or even extraintestinal infections [10]. There has been a recent uptick of otitis externa cases caused by *V. alginolyticus* in the Northern Hemisphere, more commonly during the summer months due to the increased seawater temperature. Since 2000, coastal water temperatures have risen by one degree Fahrenheit which has increased the abundance of these bacteria [11,12]. The treatment strategy of choice for *V. alginolyticus* species is either a combination of a third-generation cephalosporin and tetracycline or a fluoroquinolone [3]. The empiric management of otitis externa includes either topical (otic drops) ciprofloxacin-hydrocortisone or topical (otic drops) neomycin-polymyxin B-hydrocortisone [13]. Although our patient was treated empirically with penicillin and his infection resolved without any complications, conductive hearing loss and disease progression are fatal complications if left untreated [14].

## Conclusions

*V. alginolyticus* is a rare cause of otitis externa in people with recent exposure to temperate seawater. Due to its sensitivity to traditional antibiotics, the true incidence and prevalence of the disease is unknown. Recent shifts in global temperatures have resulted in elevated seawater temperatures, enabling *V. alginolyticus* species to cause infections in areas previously unaffected. Due to changes in *V. alginolyticus *prevalence, physicians should culture ear infections of patients with recent sea exposure to accurately diagnose this pathogen and treat it accordingly to avoid disease progression and complications. Furthermore, physicians can educate their patient populations on the prevention of *V. alginolyticus* by taking precautions when swimming or engaging in activities in warm coastal waters, especially if the patient has open wounds or is immunocompromised. Additionally, as coastal seawater continues to warm, the incidence of *V. alginolyticus* infections should be updated to reflect the environmental change.

## References

[1] Baker-Austin C, Oliver JD, Alam M, Ali A, Waldor MK, Qadri F, Martinez-Urtaza J (2018). Vibrio spp. infections. Nat Rev Dis Primers.

[2] Jacobs Slifka KM, Newton AE, Mahon BE (2017). Vibrio alginolyticus infections in the USA, 1988-2012. Epidemiol Infect.

[3] Kalyoussef S, Feja KN (2014). Foodborne illnesses. Adv Pediatr.

[4] Liu XF, Zhang H, Liu X, Gong Y, Chen Y, Cao Y, Hu C (2014). Pathogenic analysis of Vibrio alginolyticus infection in a mouse model. Folia Microbiol (Praha).

[5] Mukherji A, Schroeder S, Deyling C, Procop GW (2000). An unusual source of Vibrio alginolyticus-associated otitis: prolonged colonization or freshwater exposure?. Arch Otolaryngol Head Neck Surg.

[6] Zhou K, Tian KY, Liu XQ, Liu W, Zhang XY, Liu JY, Sun F (2021). Characteristic and otopathogenic analysis of a Vibrio alginolyticus strain responsible for chronic otitis externa in China. Front Microbiol.

[7] Schmidt U, Chmel H, Cobbs C (1979). Vibrio alginolyticus infections in humans. J Clin Microbiol.

[8] Centers for Disease Control and Prevention (CDC) (2024). Estimated burden of acute otitis externa-United States, 2003-2007. MMWR Morb Mortal Wkly Rep.

[9] Fu K, Li J, Wang Y, Liu J, Yan H, Shi L, Zhou L (2016). An innovative method for rapid identification and detection of Vibrio alginolyticus in different animal models. Front Microbiol.

[10] Duarte MJ, Kozin ED, Bispo PJ, Mitchell AH, Gilmore MS, Remenschneider AK (2018). Methicillin-resistant Staphylococcus aureus in acute otitis externa. World J Otorhinolaryngol Head Neck Surg.

[11] (2024). Extended reconstructed sea surface temperature ERSST. https://www.ncei.noaa.gov/products/extended-reconstructed-sst.

[12] Gildas Hounmanou YM, Engberg J, Bjerre KD (2023). Correlation of high seawater temperature with Vibrio and Shewanella infections, Denmark, 2010-2018. Emerg Infect Dis.

[13] Goguen L, Durand M (2024). External otitis in adults: treatment. https://www.uptodate.com/contents/external-otitis-in-adults-treatment.

[14] Goguen L (2024). External otitis: pathogenesis, clinical features, and diagnosis. https://www.uptodate.com/contents/external-otitis-pathogenesis-clinical-features-and-diagnosis.

